# Supplementary material for: Scalable long read self-correction and assembly polishing with multiple sequence alignment
Source: Sci Rep. 2021 Jan 12;11:761. doi: 10.1038/s41598-020-80757-5 (PMC7804095; doi:10.1038/s41598-020-80757-5)
Supplement: Supplementary file 1 — Supplementary Information. [file 41598_2020_80757_MOESM1_ESM.pdf]

Scalable long read self-correction and assembly polishing with multiple sequence alignment

Supplementary Materials

Pierre Morisse [1,*], Camille Marchet [2], Antoine Limasset [2], Thierry Lecroq [3] and Arnaud Lefebvre [3]

[1]Univ Rennes, Inria, CNRS, IRISA, F-35000 Rennes, France
[2]Univ. Lille, CNRS, UMR 9189 - CRIStAL, F-59000 Lille, France
[3]Normandie Univ, UNIROUEN, LITIS, 76000 Rouen, France

	Without segmentation	With segmentation
Number of bases	214,836,958	215,702,126
Error rate (%)	0.3394	0.1915
Recall (%)	99.9711	99.9790
Precision (%)	99.6659	99.8115
Runtime	5 h 31 min	7 min
Memory (MB)	750	675

Table S1: Comparison of the results produced by CONSENT, with and without our segmentation stategy, as reported by ELECTOR. Using the segmentation strategy allows a 47x speed-up, while producing a slightly higher quality correction.

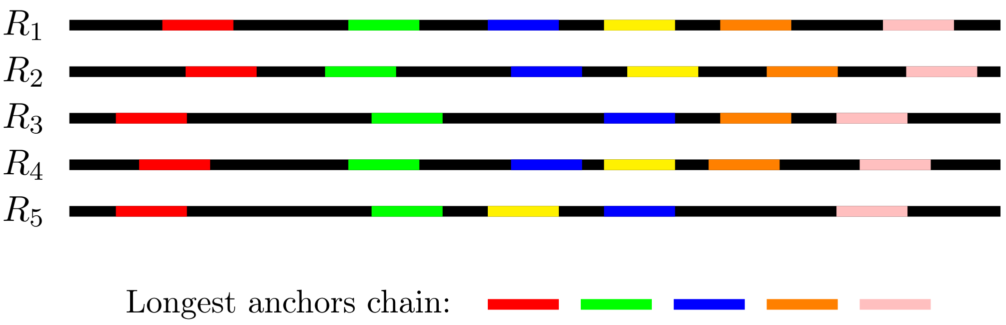

Figure S1: **Computation of the longest anchors chain for a set of sequences.** Here, we compute this chain with the second property set to $T = 3$, to simplify the figure. The longest chain computed here is thus $S =$ (red, green, blue, orange, pink). If we only considered the four first sequences, the chain $S' =$ (red, green, blue, yellow, orange, pink), which is long than S could be selected. Indeed, although R_3 does not contain the yellow anchor, S' follows the two properties in sequences R_1, R_2, and R_4. However, in R_5, the yellow anchor appears before the blue anchors, and thus invalidates the first property. S' thus cannot be selected as the longest chain for the set of sequences R_1-R_5, and S is chosen instead. Moreover, R_5 does not contain the whole set of anchors that appear in the longest anchor chain, S (orange anchor is missing). As a result, S_5 will be filtered out during the MSA and consensus computation step.

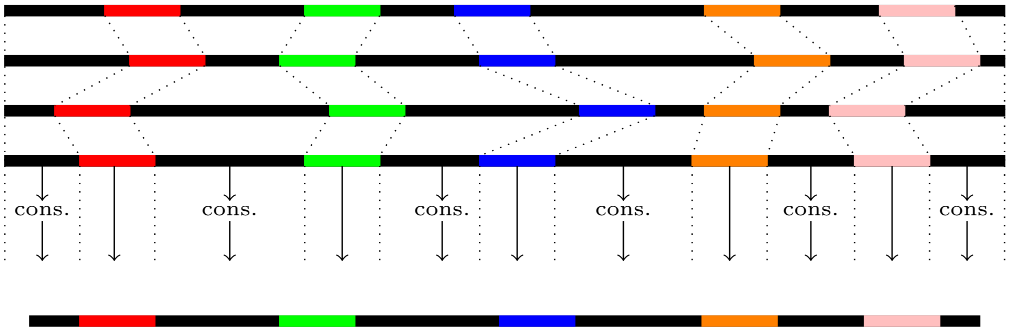

Figure S2: **Segmentation strategy for consensus computation of a window.** Anchors defining the longest anchors chain are used in order to segment the MSA and consensus computation. Local, independent consensus are thus computed on subsequenes delimited by these anchors. These local consensus, along with the anchors, are then concatenated, in order to rebuild to global consensus, at the scale of the original sequences length.

Dataset	Number of reads	Average length (bp)	Error rate (%)	Coverage	Accession
**Simulated PacBio data**					
E. coli 30x	16,959	8,235	12.29	30x	N/A
E. coli 60x	33,918	8,211	12.28	60x	N/A
S. cerevisiae 30x	45,198	8,216	12.28	30x	N/A
S. cerevisiae 60x	90,397	8,204	12.29	60x	N/A
C. elegans 30x	366,416	8,204	12.28	30x	N/A
C. elegans 60x	732,832	8,220	12.28	60x	N/A
**Real PacBio data**					
S. cerevisiae	121,640	8,900	15.37	89x	SRR9617898
**Real ONT data**					
D. melanogaster	1,327,569	6,828	14.55	63x	SRX3676783
H. sapiens[1,2] (chr 1)	1,075,867	6,744	17.60	29x	PRJEB23027
H. sapiens[2]	15,243,243	7,411	17.26	35x	PRJEB23027

Table S2: Description of the long reads datasets used in our experiments.
[1] Only reads from chromosome 1 were used.
[2] We used release 4, available at https://github.com/nanopore-wgs-consortium/NA12878/blob/master/nanopore-human-genome/rel_3_4.md.

Reference organism	Strain	Reference sequence	Size (Mbp)
E. coli	K-12 substr. MG1655	NC_000913	4.6
S. cerevisiae	S288C	NC_0011{33-48} - NC_001224	12.2
C. elegans	Bristol N2	GCA_000002985.3	100
D. melanogaster	BDGP Release 6	ISO1 MT/dm6	144
H. sapiens (chr 1)	GRCh38	NC_000001	249
H. sapiens	GRCh38	NC_000001 - NC_000024	3,257

Table S3: Description of the reference sequences used in our experiments.
[1] Only chromosome 1 was used.

Dataset	Corrector	Number of bases (Mbp)	Average length (bp)	Error rate (%)	Recall (%)	Precision (%)	Runtime	Memory (MB)
E. coli 30x	Original	140	8,235	12.2862	_	_	N/A	N/A
	LoRMA	2	201	1.1962	99.9209	98.8208	10 min	32,155
E. coli 60x	Original	279	8,211	12.2788	_	_	N/A	N/A
	LoRMA	171	886	0.1285	99.9865	99.8743	1 h 39 min	31,682
S. cerevisiae 30x	Original	371	8,216	12.283	_	_	N/A	N/A
	LoRMA	14	248	2.1640	99.8351	97.8564	46 min	31,899
S. cerevisiae 60x	Original	742	8,204	12.2886	_	_	N/A	N/A
	LoRMA	443	856	0.2225	99.9785	99.7812	5 h 25 min	31,828
C. elegans 30x	Original	3,006	8,204	12.2806	_	_ N/A	N/A	
	LoRMA	33	215	3.6960	99.7269	96.3449	8 h 19 min	31,827
C. elegans 60x	Original	6,024	8,202	12.2825	_	_	N/A	N/A
	LoRMA	781	337	0.6446	99.9547	99.3649	31 h 04 min	32,104

Table S4: Metrics output by ELECTOR on the simulated PacBio datasets, for the error correction with LoRMA. Runtime and memory consumption are reported for the whole correction pipeline. To underline the aggressive splitting of the reads performed by LoRMA, we also report the average length of the reads, in addition to ELECTOR's metrics.

On the 30x datasets, LoRMA performed worse than all the other methods, displaying 4x to 11x lower numbers of bases. This can be explained by the fact that it requires deep long-read coverage, as it is the only method purely relying on a k-mer strategy, thus fully avoiding overlaps computation. Indeed, LoRMA's number of bases was much higher, and comparable to that of other methods, on the 60x datasets. However, it produced corrected reads displaying extremely short average length, barely reaching more than 800 bp on the 60x coverage datasets, and reaching less than 300 bp on the 30x coverage datasets. As for memory consumption, LoRMA consumed 32 GB, the maximum available amount of memory, on all the datasets.

Dataset	Corrector	Number of reads	Number of bases (Mbp)	N50 (bp)	Aligned reads (%)	Alignment identity (%)	Genome coverage (%)	Runtime	Memory (MB)	
S. cerevisiae	Original	Original	121,640	1,083	12,048	96.27	84.63	99.65	N/A	N/A
	LoRMA	114,354	759	1,627	98.18	98.15	97.50	4 h 22 min	43,525	
D. melanogaster	Original	1,327,569	9,064	11,853	85.52	85.43	98.47	N/A	N/A	
	LoRMA	1,125,279	6,386	669	97.05	98.47	94.76	23 h 51 min	65,536	
H. sapiens (chr 1)	Original	1,075,867	7,256	10,568	88.24	82.40	92.46	N/A	N/A	
	LoRMA	737,198	1,247	186	96.50	97.83	28.62	13 h 07 min	50,435	

Table S5: Statistics of the real long reads, before and after correction with LoRMA. Runtime and memory consumption are reported for the whole correction pipeline.

In terms of alignment identity, LoRMA outperformed all the other tools. However, the N50 of the reads was extremely short, and did not even reach 700 bp, which is consistent the short read lengths observed in Supplementary Table S3. This confirms the fact that LoRMA tends to aggressively split the reads, and only manage to correct small portions of them. This small N50 thus explains the high alignment identities, by the fact that LoRMA tends not to correct complex regions of the reads. As a result, LoRMA also displayed the lowest genome coverage among all the tools, especially on the *H. sapiens* dataset, were the genome coverage was less than 30%. Assembly result for LoRMA corrected reads are thus not presented, since Miniasm could not manage to perform assembly with these reads.

1 Software versions and command lines

1.1 Canu

We used Canu v2.0. The following command lines were used.

- For PacBio data: `./canu -correct -p ResultsPrefix -d ResultsDirectoy genomeSize=exactSizeOfTheGenome -pacbio-raw rawLongReads.fasta -stopOnReadQuality=false -corOutCoverage=300 -useGrid=false`
- For ONT data: `canu -correct -p ResultsPrefix -d ResultsDirectory genomeSize=exactSizeOfTheGenome -nanopore-raw rawReads.fasta -corOutCoverage=300 -useGrid=false`

1.2 Daccord

Daccord requires to run a series of scripts and other programs in order to run. We provide the versions of each subprogram and the complete suite of command lines below. ConverToPacBio_q2a.py, fasta2DB, and DBsplit are provided within the DAZZ_DB suite.

1.2.1 Versions

- DAZZ_DB version July 17, 2013.
- DALIGNER version April 10, 2016.
- Daccord version v0.0.10.

1.2.2 Command lines

The following command lines were used for both PacBio and ONT data.

- `./ConverToPacBio_q2a.py rawReads.fasta` (This command creates, by default, a formatted file called "LR.fasta", which we use as an input for the following steps)
- `./fasta2DB readsDb LR.fasta`
- `./DBsplit -x14 readsDb.db`

- ./daligner -T16 readsDb.db readsDb.db
- ./daccord -t16 readsDb.readsDb.las readsDb.db > correctedReads.fasta

1.3 FLAS

We used FLAS commit 053c19b. The following command line was used for both PacBio and ONT data.

- python runFLAS.py rawReads.fasta

1.4 LoRMA

We used LoRMA v0.4. The following command line was used for both PacBio and ONT data.

- ./lorma.sh -s -threads 16 rawReads.fasta

1.5 MECAT

We used MECAT commit d04bfa8. The following command lines were used.

1.5.1 For PacBio data

- ./mecat2pw -j 0 -d rawReads.fasta -w . -t 16 -o rawReads.fasta.can
- ./mecat2cns -i 0 -t 16 rawReads.fasta.can rawReads correctedReads.fasta

1.5.2 For ONT data

- ./mecat2pw -j 0 -d rawReads.fasta -o candidates.txt -w . -t 28 -x 1
- ./mecat2cns -i 0 -t 28 -x 1 candidates.txt rawReads.fasta correctedReads.fast

1.6 RACON

We used RACON v1.3.2. The following command line was used for both PacBio and ONT data.

- racon -t28 rawReads.fasta rawReadsXrawAssemblyOverlaps.paf rawAssembly.fasta > polishedAssembly.fasta

1.7 QUAST-LG

We used QUAST-LG v5.0.2. The following command lines were used.

1.7.1 For the *E. coli* 60x dataset

- `./quast.py -o ResultsDirectory -r referenceGenome.fasta assembly.fasta -t16 -min-identity 80`

1.7.2 For the *S. cerevisiae* 60x and *S. cerevisiae* real datasets

- `./quast.py -o ResultsDirectory -r referenceGenome.fasta assembly.fasta -t16 -min-identity 80 -fragmented`

1.7.3 For the*C. elegans* 60x, *D. melanogaster*, *H. sapiens* (chr 1), and *H. sapiens* complete datasets

- `./quast.py -o ResultsDirectory -r referenceGenome.fasta assembly.fasta -t16 -min-identity 80 -large -fragmented`

1.8 Miniasm

We used Miniasm v0.3-r179. The following command line was used for the assembly of both PacBio and ONT data.

- `./miniasm -f rawReads.fasta RawReadsxRawReadsOverlaps.fasta`

1.9 Minimap2

We used Minimap2 v2.17-r974-dirty. The following command lines were used.

1.9.1 For PacBio data correction

- `./minimap2 -dual=yes -PD -no-long-join -w5 -g1000 -m30 -n1 -t16 -I1G rawReads.fasta rawReads.fasta > Overlaps.paf`

1.9.2 For ONT data correction

- `./minimap2 -dual=yes -PD -no-long-join -k15 -w5 -m100 -g10000 -r2000 -max-chain-skip 25 -t28 -I1G rawReads.fasta rawReads.fasta > Overlaps.paf`

1.9.3 For assembly polishing

- `./minimap2 -dual=yes -PD -no-long-join -w5 -g1000 -m30 -n1 -t16 -I1G rawAssembly.fasta rawReads.fasta > Overlaps.paf`

1.9.4 For PacBio data assembly (prior to Miniasm)

- `./minimap2 -x ava-pb -t28 reads.fasta reads.fasta > Overlaps.paf`

1.9.5 For ONT data assembly (prior to Miniasm)

- `./minimap2 -x ava-ont -t28 reads.fasta reads.fasta > Overlaps.paf`
